# Inhibition of ferroptosis alleviates high-power microwave-induced myocardial injury

**DOI:** 10.3389/fcvm.2023.1157752

**Published:** 2023-04-24

**Authors:** Yu Wang, Yan Lu, Wen Chen, Xiaohua Xie

**Affiliations:** Department of Comprehensive of the Second Medical Center & National Clinical Research Center for Geriatric Diseases, Chinese PLA General Hospital, Beijing, China

**Keywords:** high-power microwave (HPM), ferroptosis, myocardial injury, GPX4, tanshinone llA

## Abstract

**Background:**

The use of high-power microwave (HPM) in our daily live is becoming more and more widespread, but the safety has also caused our concern. And ferroptosis is a newly discovered modality that can regulate cell death in recent years. The aim of our study was to demonstrate whether ferroptosis is an important cause of myocardial injury caused by HPM. And whether myocardial injury caused by HPM can be alleviated by inhibiting ferroptosis.

**Methods:**

We verified the extent of myocardial damage by different doses of HPM through *in vivo* and *in vitro* assays, respectively. In addition, GPX4 was knocked down and overexpressed in cardiac myocytes to verify the altered sensitivity of cardiac myocytes to HPM. Finally, the therapeutic effect of Fer-1 and tanshinoneIIA on myocardial injury caused by HPM was verified in *in vivo* and *in vitro* assays.

**Results:**

We found that cardiac tissue and cardiomyocyte injury in mice gradually increased with increasing HPM dose, while ferroptosis markers were consistent with the injury trend. Gpx4 had an important role in ferroptosis in cardiomyocytes caused by HPM. Finally, tanshinoneIIA and Fer-1 could attenuate the damage of cardiac tissues and cardiomyocytes caused by HPM.

**Conclusions:**

In conclusion, our study found that ferroptosis, a novel mode of cell death, is present in myocardial injury caused by HPM. Moreover, tanshinone, a drug already in clinical use, can significantly reduce myocardial injury caused by HPM, which is promising to provide new therapeutic ideas for myocardial injury caused by HPM.

## 1. Introduction

As times develop and the wireless applications continue to make progress, the use of microwaves becoming more and more widespread in our lives (1). Microwaves are non-ionizing radiation electromagnetic waves and are increasingly used in domestic, industrial, medical instruments and communication applications (2). Even high-power microwave (HPM) can be used to detect potential improvised explosive devices (IEDs) in some security works (3).

While these applications have brought convenience to our lives, the safety of this technology has raised more concerns as people are exposed to microwaves more frequently (4). Some investigations have found that certain intensities of microwave radiation, especially HPM, can cause some degree of damage to several organs of the body (2, 5, 6). In one study, rats were placed in a microwave irradiation environment at a frequency of 1,800 MHZ for 5 weeks. It was found that the myocardial fibers in the rats were widened and disorganized, resulting in a disruption of myocardial contractile function (7). Microwave irradiation could have variable impacts on the cardiovascular system (8, 9). A study showed that myocardial enzymes and ion homeostasis were disrupted in rats exposed to microwave irradiation at different average power densities: 0, 2.5, 5 and 10 mW/cm^2^, indicating that microwave irradiation can cause some damage to the myocardium (10). When the average power of microwave irradiation was 11 mW/cm^2^ it caused cardiac arrhythmias, damage to the myocardial conduction system and the formation of myocardial fibrosis in rhesus monkeys (11).

There are two types of cell death: accidental cell death(ACD) and regulated cell death(RCD) (12). In 2012, Stock well et al. formally proposed a novel RCD—ferroptosis (13). Ferroptosis is a phenomenon manifested by rupture of the outer mitochondrial membrane, reduction or disappearance of cristae, and membrane denseness (13–15). Abundant intracellular iron promotes lipid peroxidation *via* the production of ROS as a result of the iron-dependent Fenton reaction (13). Therefore, the essence of ferroptosis is that intracellular iron-dependent peroxidation of lethal lipids occurs (16).

It is interesting to note that some studies have found in their studies of microwave radiation that damage to organisms or cells is based on a breakdown in the balance between free radical production and scavenging (17, 18). Up to now, ferroptosis has been associated with pathological changes in myocardial ischemia-reperfusion, associated with DOX-induced cardiomyopathy (19, 20).

It has been reported that many natural compounds derived from nature can ameliorate pathological states by regulating ferroptosis (21–24). Traditional Chinese medicine (TCM) compatibility is a jewel of TCM. Ever the advent of Shennong Ben Cao Jing, the compatibility of TCM has been advocated and standardized in clinical application. With the development of TCM, more and more attention has been paid to the application of compatibility of TCM. Tanshinone IIA (TSA) is a representative of the fat-soluble components of Salvia miltiorrhiza Bunge, and is also the main active component of Salvia miltiorrhiza Bunge as well as the basis for exerting pharmacological activity. It has anti-inflammatory, bacteriostatic, anti-myocardial hypertrophy, and improves coronary circulation. TSA also can regulate intracellular redox status and is widely used in the treatment of cardiovascular disease. In addition, it has been found that TSA can protect endothelial cells by alleviating ferroptosis (25, 26).

Although some studies have already suggested that S-band microwaves cause structural abnormalities and impaired function in the myocardium (27), no studies have been done to report whether there is ferroptosis in myocardial damage caused by microwave radiation. In this study, we analyzed whether mice subjected to different HPM conditions suffered damage to myocardium in which there was RCD—ferroptosis. Furthermore, we explored whether TSA, a naturally occurring chemosynthetic extract, plays a role in regulating ferroptosis in a model of myocardial damage induced by microwave irradiation, thereby alleviating cardiac function.

## 2. Materials and methods

### 2.1. Animals

The male Institute of Cancer Research (ICR) mice (18–20 g, 6–8-week-old) were purchased from the Wuhan Shubeili Biology science and technology. The animals were fed by the Laboratory Animal Center of the Academy Military Medical Sciences. The animal room conditions maintained on temperature 20–26°C; daily temperature difference: ≤4°C; relative humidity-70%; alternating day and night light-dark time: 12/12 h. The experimental protocols were approved by theAcademy of Military Medical Sciences, ethical No. 2022-x18-21.

### 2.2. HPM-irradiated mice and therapy

The microwave source is a courtesy of the Pathology Research Unit, Institute of Radiological Medicine, Academy of Military Medical Sciences, capable of generating pulsed microwaves in the S-band. The mice were exposed to radiation with an average power density of 0, 10, 20 and 30 mW/cm^2^ for 30 min every time, once a week for two weeks.

Mice in the treatment group respectively accepted 0 (Control group), 30 mW/cm^2^ (HPM group) radiation. The other two groups of mice were given Tanshinone IIA sulfonate sodium (MCE, HY-N1370) and Ferrostatin-1 (Synonyms: Fer-1, Sigma, SML0583) after receiving 30 mW/cm^2^ irradiation. Both groups were administered from the day before the first irradiation for 15 consecutive days at a frequency of once a day. Equal volumes of PBS were given to the control and irradiated groups, Tanshinone was administered at 20 mg/kg and 1.5 mg/kg to the Fer-1 group.

### 2.3. Basic information of mice

Weigh mice for weight, heart, lung, and tibia length. Measure the anal temperature of mice before and after irradiation using animal thermometer.

### 2.4. Echocardiographic measurements

Cardiac function was examined by placing a two-dimensional M-mode transthoracic echocardiogram on an ultrasound instrument (Vinno6, China). The frequency used for mouse heart ultrasound is 23 MHZ, and the range is 10–23 MHz. The probe model is shown at the probe plug location, and X10-23l is the model number of this probe. Mice were anesthetized using 2% isoflurane by inhalation and then placed in the supine position to capture images. Parameters of cardiac function, including left ventricular ejection fraction (LVEF), partial shortening (FS), left ventricular posterior wall dimensions (LVPWd), left ventricular posterior wall systolic thickness (LVPWs), Left Ventricular Internal Dimension systole (LVIDs) and Left Ventricular Internal Diastolic Dimension (LVIDd).

### 2.5. Elisa

Weigh the mouse heart (mg) : volume (ul) = 1 : 9 ratio by adding 9 times the volume of homogenization medium (0.9% saline). The homogenate was prepared mechanically under ice-water bath conditions to make 10% homogenate. Then 2,500–3,000 rpm, centrifuged for 10 min and the supernatant was taken for determination. Add diluted biotinylated antibody: IL-6 (ThermoFisher, catalog number 88-7064), IL-1 beta (ThermoFisher, catalog number 88-7013), TNF alpha (ThermoFisher, catalog number 88-7324) working solution to each well, then diluted enzyme conjugates working solution to each well. Ultimately on the enzyme labeling instrument, at 450 nm, the O.D. of each well was measured after the blank control pore was zeroed.

### 2.6. Detection of CAT, MDA, SOD, GSH-Px in myocardial tissue

Myocardial tissue was sampled in the same way as in method 5. OD values were determined using theCatalase (CAT) assay kit (Visible light) (Nanjing Jiancheng Bioengineering Institite, catalog numberA007-1-1) at 405 nm absorbance. The formula for CAT is:

The dynamism of CAT in the organisation (*U/mgprot*) = (Control OD − Measurement OD)*271* $\frac{1}{60*\mathrm{Sampling}\;\mathrm{volume}}$ ÷ Protein concentration of the sample to be tested (mgprot/ml)

Malondialdehyde (MDA) assay kit (TBA method) (Nanjing Jiancheng Bioengineering Institite, catalog numberA003-1-2) was used at 532 nm to test OD values. The formula for MDA is:

The dynamism of MDA in the organisation (nmol/mgprot) = $\frac{\mathrm{Measurement}\;\mathrm{OD}-\mathrm{Control}\;\mathrm{OD}}{\mathrm{Standard}\;\mathrm{OD}-\mathrm{Blank}\;\mathrm{OD}}$ * Standard concentration (10 nmol/ml) ÷ Protein concentration of the sample to be measured (mgprot/ml)

Total Superoxide Dismutase (T-SOD) assay kit (Hydroxylamine method) (Nanjing Jiancheng Bioengineering Institite, catalog numberA001-1-2) detect OD at 550 nm. The formula for SOD is:

SOD viability in tissues (U/mgprot) = $\frac{\mathrm{Control}\;\mathrm{OD}-\mathrm{Measurement}\;\mathrm{OD}}{\mathrm{Control}\;\mathrm{OD}}$ ÷ 50% * $\frac{\mathrm{Total}\;\mathrm{volume}\;\mathrm{of}\;\mathrm{reaction}\;\mathrm{solution}}{\mathrm{Sampling}\;\mathrm{volume}(\mathrm{ml})}$ ÷ Protein concentration of the sample to be tested (mgprot/ml)

Glutathione Peroxidase (GSH-PX) assay kit (Colorimetric method) (Nanjing Jiancheng Bioengineering Institite, catalog numberA005-1-2) was used at 412 nm to test OD values. The formula for MDA is:

GSH-PX enzymatic activity (U/mgprot) = $\frac{\mathrm{Control}\;\mathrm{OD}-\mathrm{Measurement}\;\mathrm{OD}}{\mathrm{Standard}\;\mathrm{OD}-\mathrm{Blank}\;\mathrm{OD}}$ * Standard concentrations (20 μmol/l) * Dilution times ÷ Reaction time ÷ Concentration of the protein sample to be measured

### 2.7. Measurement of plasma Fe and cardiac troponin I (cTnI)

Blood was collected from the posterior orbital vein of mice and centrifuged at 3,000 r for 15 min using 4°C centrifuge and the supernatant was removed for testing. Fe concentration using the iron determination test kit (Changchun Huili Biotechcol TD, catalog numberC016). And we used ELISA Kit for Cardiac Troponin I (cTnI) (Cloud-Clone CORP. Wuhan,catalog numberSEA478Mu) to quantify the amount of TNNI3 in mouse serum.

### 2.8. He staining

The mouse hearts were removed and immersed in 4% paraformaldehyde for 48 h, followed by paraffin embedding. Sections were stained with hematoxylin and eosin staining. Finally, the morphological changes of the heart were photographed under a light microscope at 200×. Myocardial injury was scored with reference to the following criteria: (1) Absence of inflammatory infiltration in the heart was scored as 0; (2) Presence of small inflammatory lesions of less than 100 inflammatory cells in the heart was scored as 1; (3) Presence of large inflammatory lesions of greater than 100 inflammatory cells in the heart was scored as 2; (4) Total area of inflammatory infiltrates greater than 10% but less than 30% of the total longitudinal area of the heart was scored as 3; (5) Total area of inflammatory infiltrates greater than 30% of the total longitudinal area of the heart was scored as 4 (5) Total area of inflammatory infiltrates greater than 30% of the total longitudinal area of the heart, assessed as 4.

### 2.9. Western blot

Cardiac tissue or myocardial cells are lysed using radioimmunoprecipitation (RIPA) lysis buffer (Beyotime, catalog number P0013D) on ice. 20 ug of cellular proteins were loaded in each well and separated by sodium dodecyl sulfate polyacrylamide gel electrophoresis (SDS-PAGE). Overnight incubation with the following antibodies: GAPDH (1:1,000, abcam, ab8245), PTGS2 (1:1,000, CST, #37843), GPX4 (1:1,000, CST, #52455), HMOX1 (1:1,000, CST, #43966), ACSL4 (1:1,000, Servicebio, GB113871), SLC7A11 (1:1,000, Servicebio, #GB115276). After washing the membranes 3 times, the membranes were incubated with horseradish peroxidase-coupled secondary antibodies for 2 h (1:5,000; Santa). Finally, densitometric analysis was performed using image acquisition and analysis software (Bio-Rad).

### 2.10. qRT-PCR (real-time quantitative PCR)

Total RNA isolation was performed using Trizol reagent (Beyotime, R0016). And using NanoDrop-2000 spectrophotometer (Thermo Fisher) assessed the RNA quality and concentration. Followed by reverse transcription of 500 ng of total RNA into cDNA using the Maxima First Strand cDNA Synthesis Kit (Thermo Fisher) in a reaction volume of 20 μl. 100 ng of cDNA was used for qRT-PCR using SYBR Premix Ex Taq II (Takara Biomedical Technology) and gene-specific primer sets. β-actin was used as an internal control. Relative expression of genes was normalized to β-actin using the 2^−ΔΔCt^ method, where ΔΔCt = (*C_t_* _target gene_ − *C_t_*_ β-actin_) treated cells − (*C_t_* _target gene_ − *C_t_* _GAPDH_) control cells. We list the primers as follows: GPX4: (F)GCACATGGTCTGCCTGGATAAG, (R)TCTTGATTACTTCCTGGCTCCTG; PTGS2: (F)GAAATATCAGGTCATTGGTGGAGA, (R)ATGCTCCTGCTTGAGTATGTCG; β-actin: (F)GTGACGTTGACATCCGTAAAGA, (R)GTAACAGTCCGCCTAGAAGCAC.

### 2.11. Immunofluorescence

After perfusion of the mouse hearts, the hearts were placed in cold 4% paraformaldehyde for 24 h. The tissue was transferred to 30% sucrose solution, dehydrated for 3 days and then frozen sectioned. After blocking, the sections were incubated overnight with primary antibody: CD45, CD68 and Invitrogen fluorescent secondary antibody (1:1,000) was added. After DAPI re-staining nucleation, the slices were sealed. Finally, a confocal microscope was used to take pictures.

### 2.12. Extraction and culture of adult mouse cardiomyocytes

Prepare two 40 ml tubes of Kreb's Ringer (KR) solution in the following ratios: NaCl 2.0454 g/L, potassium dihydrogen phosphate 0.1619 g/L, potassium chloride 0.3541 g/L, disodium hydrogen phosphate 5.7302 g/L, sucrose 45.8669 g/L, glucose 1.9817 g/L. sodium bicarbonate 2.1003 g/L, HEPEs 2.3831 g/L and preheated in a 44°C water bath. After preheating, the pH of the KR solution was adjusted to 7.4 with NaOH. 40 mg of type II collagenase was weighed and added to one of the perfusion tubes to prepare 1 mg/ml of heart digest. The KR fluid and digestive fluid were poured into the infusion tube successively, so that the first half of the fluid flowing out of the tube was KR fluid to flush out the blood from the heart chambers, and the second half was digestive fluid. After exposing the mouse heart, heparin sodium solution was pushed rapidly through the right auricle to remove the remaining blood from the heart and prevent it from clotting. The gavage needle was inserted retrogradely from the broken end of the aortic arch to the top of the aortic valve and perfused until the heart gradually expanded and became transparent. The right and left auricles, right ventricle and septum were carefully removed with ophthalmic scissors, leaving only the left ventricle, which was cut into pieces and blown with a 2 ml bacchus dropper until the cardiomyocytes were dispersed. The cell suspension was filtered through a 200 mesh cell strainer. The filtered cells were placed in a 15 ml centrifuge tube (containing KR fluid) and settled naturally.

After the KR solution was discarded, the cells were resuspended by adding Recalcitrant Solution 1 and then settled for 15 min. The cardiomyocytes were then recalcitrated to their physiological state by adding Recalcitrant Solution 2 and Recalcitrant Solution 3 in that order. The cardiomyocytes were then resuspended with complete medium for culture. Place in an incubator 37°C and 5% CO_2_ to incubate.

**Table:** Complete medium formulation

Element	Storage concentration	volume (ml)	Final concentration
MEM	–	96	–
BSA	5%	2	0.1%
ITS	100×	1	1×
BDM	1 mol/l	1	10 mm/l
CD lipid	100×	1	1×
p/s	100×	1	1×

**Table:** Calcium complex solution formulation

Solution	Recalcitrant Solution 1	Recalcitrant Solution 2	Recalcitrant Solution 3
KR solution	15	10	5
Complete medium formulation	5	10	15

### 2.13. Irradiated cardiomyocytes

Place cardiomyocytes in a 6-well plate at a density of 2.5 × 10^5^/well. When the cells are attached to the wall and in a stable state, they were irradiated for 15 min according to HPM 0, 10, 20, 30 mW/cm^2^. Cells were collected after 48 h of irradiation for subsequent experiments.

The treatment cells were divided into 4 groups: control; HPM, irradiated for 15 min on HPM 30 mW/cm^2^; HPM + Tanshinone, Tanshinone IIA (1 μmol/l) was started 12 h before irradiation and cells were collected for subsequent experiments until 48 h after irradiation; HPM + Fer-1, Fer-1 (0.1 μmol/l) was started 12 h before irradiation and cells were collected for subsequent experiments until 48 h after irradiation.

### 2.14. Cell viability

Cells were spread in 96-well plates at 5,000 cells/well and irradiated at different doses after they were walled up. Add MTS:PMS = 20:1 to the cells 4 h in advance, following 20 ul/well. The OD values were measured at 490 nm on an enzyme marker.

### 2.15. Flow cytometry for ROS detection

Cardiomyocytes were spread in 6-well plates at a density of 2.5 × 10^5^/well and irradiated with HPM or use of medication. Cells were collected 48 h after irradiation for ROS determination. Reactive Oxygen Species Assay Kit (Servicebio, catalog numberG1706-100T) was used to dectet ROS. Add 1,000 ul/well of DCFH-DA working solution and incubate for 30 min at 37°C in a CO_2_ incubator protected from light. And then using a flow cytometer, the DCF was excited by 488 nm excitation light and emitted 530 nm fluorescence signal, which was received by the FITC channel.

### 2.16. JC-1 kit for detection of mitochondrial membrane potential

We uesd JC-1 Mitochondrial Membrane Potential Assay Kit (Servicebio, catalog numberG1515-100T) to detect mitochondrial membrane potential in cardiomyocytes. After the cells were plated in 2.5 × 10^5^/well, JC-1 staining solution was added and incubated in the incubator for 20 min. The wavelength parameters for JC-1 polymer detection by flow cytometry were Ex = 525 nm and Em = 590 nm.

### 2.17. siGPX4 and ovGPX4

SiGPX4 and ovGPX4 were obtained from Miaoling bio. The siRNA sequences used are: siGPX4#1 GTAACGAAGAGATCAAAGA; siGPX4#2 GAGGCAAGACCGAAGTAAA. pLV3-CMV-GPX4(mouse)-3xFLAG-CopGFP-Puro(Cat.#P42966) was used overexpression GPX4.

### 2.18. Statistical analysis

The data were analyzed by GraphPad Prism 9.3.1 software and presented as the means ± SEM. Two group of comparison was performed by two tailed Student's *t*-test. Multi-group's comparisons were performed by one-way ANOVA. And all statistical analyses were calculated by SPSS26.0 software. *P* < 0.05 was considered to be significant difference statistically.

## 3. Results

### 3.1. HPM causes deterioration in the basic condition of mice and myocardial damage

Compared to HPM 0 mW/cm^2^, different HPM caused mice to eat less, consume less water, and have less shiny coats. As well as a decrease in body weight, an increase in heart weight and lung weight, an increase in the ratio of heart weight and lung weight to body weight, and an increase in the ratio of heart weight and lung weight to tibia length in mice (Table 1).

**Table 1:** Body weight, heart and lung weight tibia length parameters in different HPM mice.

	HPM: 0 mW/cm^2^ (*n* = 6)	HPM: 10 mW/cm^2^ (*n* = 6)	HPM: 20 mW/cm^2^ (*n* = 6)	HPM: 30 mW/cm^2^ (*n* = 6)
Body weight (g)	19.58 ± 0.47	19.33 ± 0.15	18.22 ± 0.28	17.23 ± 0.23
Heart weight (mg)	111.28 ± 2.95	133.73 ± 1.70	163.05 ± 2.16	191.08 ± 83.15
Lung weight (mg)	144.08 ± 2.47	152.28 ± 3.87	228.43 ± 6.47	283.25 ± 1.04
Tibial length (mm)	17.50 ± 0.13	17.62 ± 0.23	17.38 ± 0.33	17.42 ± 0.12
Heart weight/body weight	5.68 ± 0.13	6.92 ± 0.11	8.95 ± 0.17	11.09 ± 4.82
Lung weight/body weight	7.36 ± 0.17	7.88 ± 0.24	12.54 ± 0.37	16.44 ± 0.23
Heart weight/tibial length	6.36 ± 0.16	7.59 ± 0.16	9.38 ± 0.15	10.08 ± 4.78
Lung weight/tibial length	8.23 ± 0.17	8.65 ± 0.24	13.14 ± 0.22	16.26 ± 0.13

**Figure 1:**
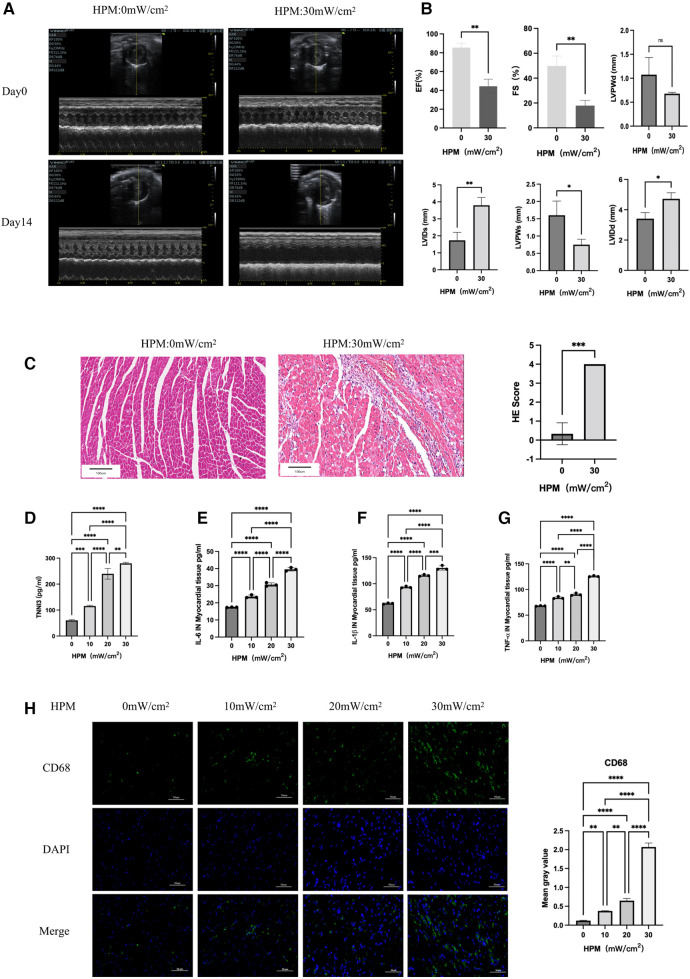
HPM causes myocardial damage in mice (*n* = 6). (**A**) Echocardiography in HPM 0 mW/cm^2^ and HPM 30 mW/cm^2^ mice pre and after HPM. (**B**) Parameters of HPM 0 mW/cm^2^ and HPM 30 mW/cm^2^ mice cardiac function, EF, FS, LVPWd, LVids, LVPWs and LVIDd. ns, *P* > 0.05; *, *P* < 0.05; **, *P* < 0.01, Student's *t*-test. (**C**) HE staining in mouse myocardial tissue. Scale bar: 100 μm. On the right, the number of inflammatory cells per mouse under the microscope is counted. Data presented are mean ± SD of microscopic myocardial injury scores. ***, *P* < 0.001, Student's *t*-test. (**D**) TNN3 concentration in mouse serum.**, P < 0.01, one-way ANOVA, *post hoc* comparisons, Tukey's test. (**E–G**) Elisa test the levels of IL-6, IL-1β and TNF-α in myocardial tissue of mice. **, P < 0.01,***, *P* < 0.001, one-way ANOVA. (**H**) Immunofluorescence technique staining of CD68, DAPI in myocardial section were conducted. On the right are the corresponding fluorescence statistics. Scale bar: 50 μm.

By comparing cardiac ultrasound in mice with HPM 0 mW/cm^2^ and HPM 30 mW/cm^2^, we found reduced myocardial contractility and reduced EF, FS, LVIDs, LVPWs and LVIDd rising (Figures 1A,B). At the same time, HE staining of myocardial sections showed that HPM resulted in large foci of inflammatory infiltration in the myocardium of mice (Figure 1C). In addition, we took blood from the posterior orbital vein of mice and measured cardiac troponin I (TNNI3) levels using an Elisa test kit. We found that cardiac troponin increased with increasing HPM (Figure 1D).

### 3.2. HPM causes increased levels of inflammation in mice

We homogenized the mouse myocardium and measured the levels of inflammatory factors in the myocardium using Elisa assay kit. We found that there was a progressive increase in IL-6, IL-1β and TNF-α in the myocardium accompanied by higher doses of HPM (Figures 1E–G).

We removed mouse hearts and performed frozen sections. Immunofluorescence staining for CD45 (Supplementary Extended Data Figure S1A) and CD68 (Figure 1H) expression levels in mouse myocardium suggested a gradual increase in CD45 and CD68 expression levels with increasing HPM.

### 3.3. HPM causes ferroptosis in mouse hearts

**Figure 2:**
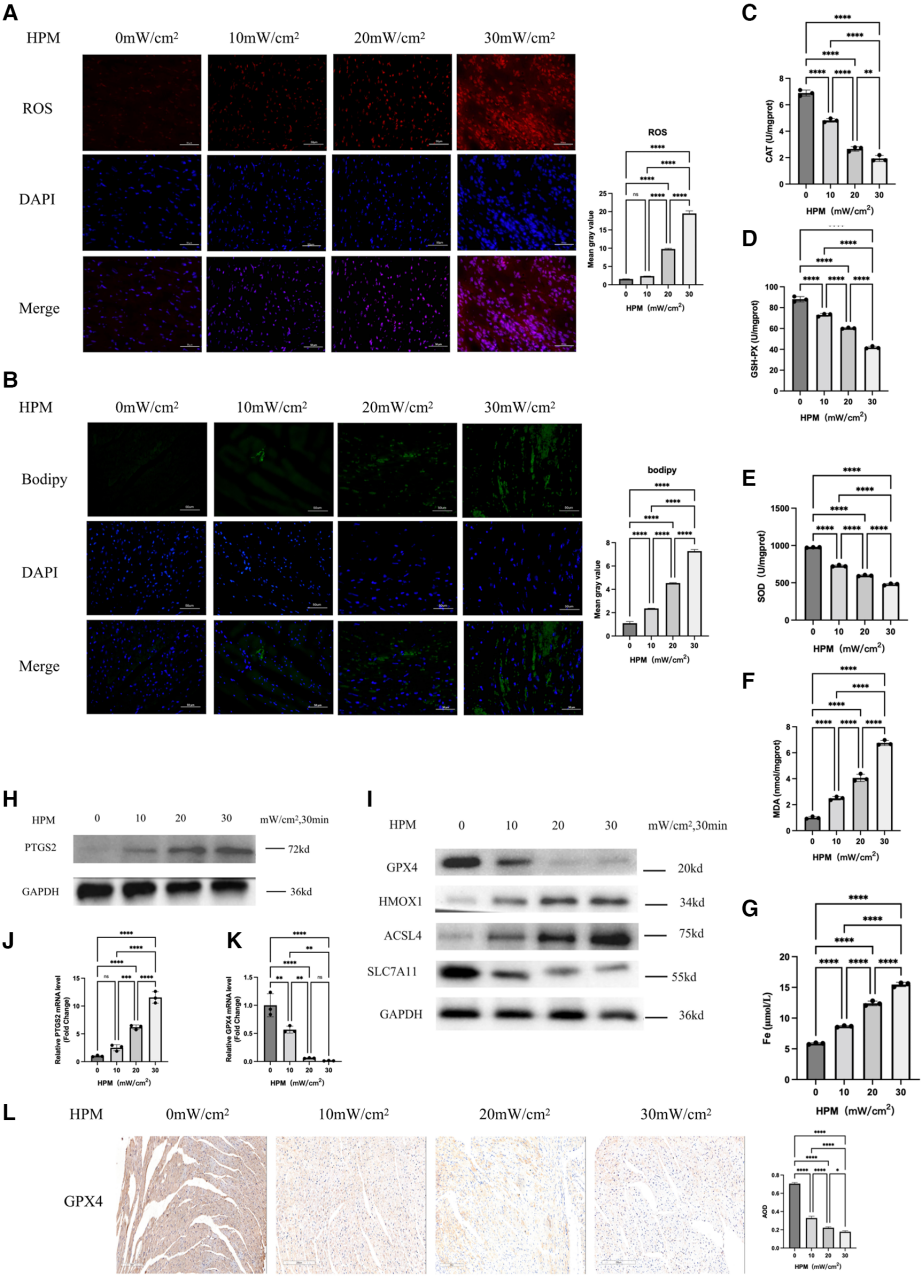
Ferroptosis occurs in myocardial tissue of HPM mice (*n* = 6). (**A,B**) Immunofluorescence technique staining of ROS, bodipy and DAPI in myocardial section were conducted and corresponding fluorescence statistics. Scale bar: 50 μm. (**C–F**) Elisa kit detect MAD, CAT, SOD and GSH of myocardial redox status in mice. (**G**) Serum Fe concentration in mice. ****, *P* < 0.0001, one-way ANOVA, *post hoc* comparisons, Tukey's test. (**H,I**) Protein levels of PTGS2, GPX4, HMOX1, ACSL4, SLC7A11 from the myocardial tissue in HPM-mice were analyzed with Western blot. (**J,K**) qRT-PCR analysis of PTGS2, GPX4 mRNA level was done in the myocardial tissue. **, P < 0.01, one-way ANOVA. (**L**) Immunohistochemistry (IHC) staining of GPX4 in myocardial tissue sections were conducted and corresponding statistics. Scale bar: 200 μm.

We examined three important redox enzymes: CAT, SOD and GSH-PX, as well as the important redox product MDA. We used an Elisa kit for the detection of myocardial redox status in mice, and we found that the activity of the three enzymes gradually decreased with increasing HPM power density, while MDA gradually increased (Figures 2C–F).

We used western blot and PCR to examine the protein and gene expression levels of the ferroptosis gold standard PTGS2 and we found that PTGS2 expression increased with increasing doses of HPM (Figure 2H). Meanwhile, immunofluorescence staining showed that Bodipy staining, a lipophilic fluorescent probe, was progressively enhanced with increasing HPM (Figure 2B).

### 3.4. Different degrees of cardiac ferroptosis occurring at different HPM doses

We used immunofluorescence to detect ROS levels and we found that ROS increased with increasing HPM (Figure 2A). At the same time, immunohistochemical detection of GPX4 expression levels in heart paraffin sections showed that GPX4 expression levels decreased with increasing HPM (Figure 2L). We also used western blot to examine the expression levels of important proteins in the ferroptosis pathway, such as GPX4, HMOX1, ACSL4 and SLC7A11 (Figure 2I).

### 3.5. HPM causes a progressive reduction in the cell viability of cardiomyocytes and the presence of ferroptosis in cardiomyocytes with reduced viability

**Figure 3:**
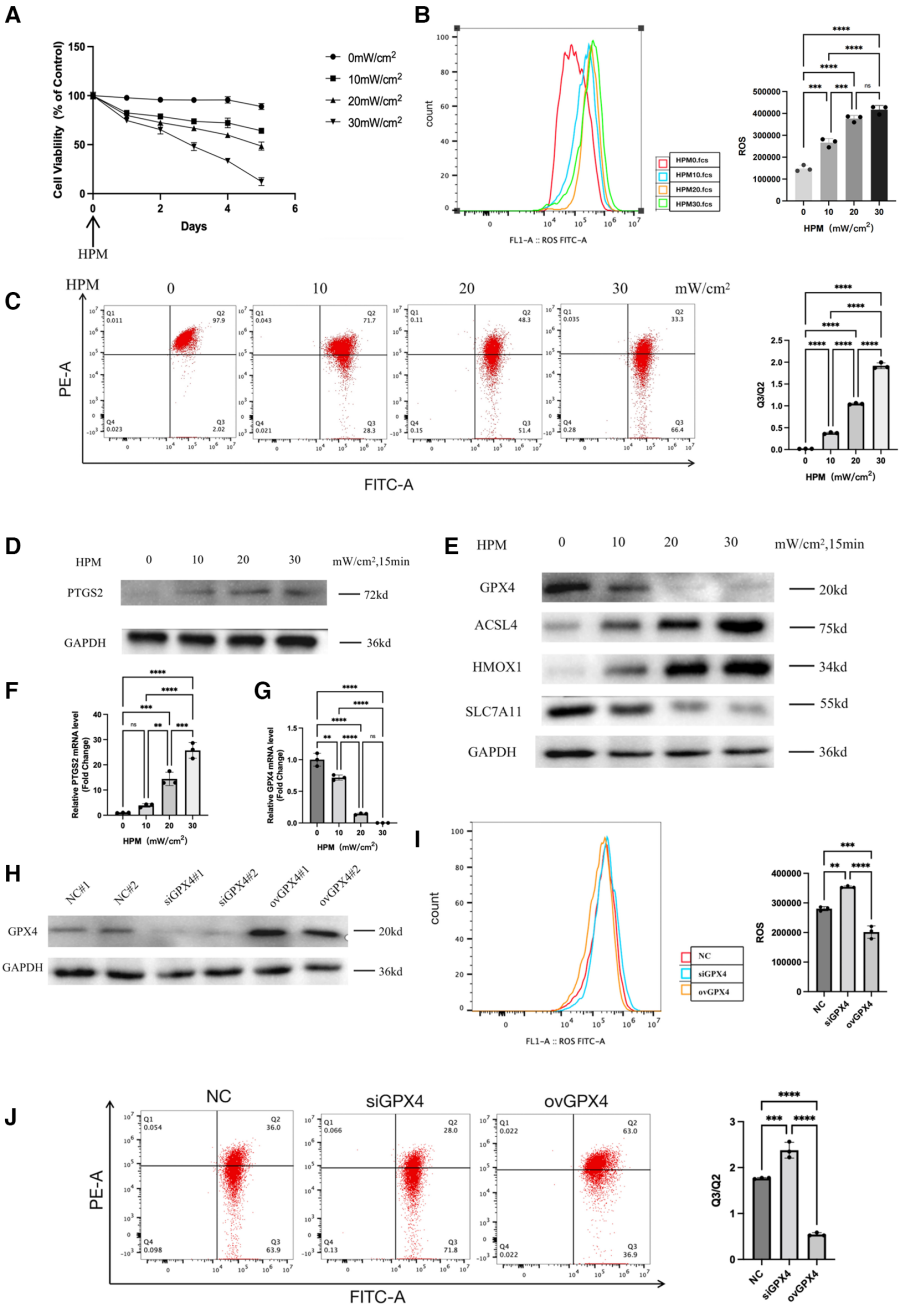
Ferroptosis occurs in the HPM cardiomyocytes. (**A**) Cardiomyocytes cells were treated with different HPM, and the cell viability was measured by MTS assay from 0 to 5 day after HPM. (**B**) ROS assay was performed in the cardiomyocytes treated with 4 concentrations of HPM for 15 min. Representative flow cytometry dot plots (left) for cardiomyocytes cells and quantitative analysis (right). Data represent mean ± SD. ns, not significant; ***, P < 0.001,****, P < 0.0001, one-way ANOVA, *post hoc* comparisons, Tukey's test. (**C**) JC-1 Mitochondrial Membrane Potential Assay Kit performed in the cardiomyocytes. (**D,E**) Protein levels of PTGS2, GPX4, HMOX1, ACSL4, SLC7A11 from the cardiomyocytes were analyzed with Western blot. (**F,G**) qRT-PCR analysis of PTGS2, GPX4 mRNA level was done in the cardiomyocytes. **, P < 0.01, one-way ANOVA. (**H**) Cardiomyocytes stably transduced with GPX4-siRNA and overexpression constructs and then subjected to Western blot. (**I,J**) ROS assay and JC-1 Mitochondrial Membrane Potential assay were performed in the control, siGPX4, ovGPX4 cardiomyocytes. Representative flow cytometry dot plots (left) and quantitative analysis (right). Data represent mean ± SD. ns, not significant; ***, P < 0.001, **, P < 0.01, one-way ANOVA, *post hoc* comparisons, Tukey's test.

We measured the viability of cardiomyocytes after HPM irradiation using MTS and found that all doses of HPM, except HPM0mW/cm^2^, resulted in a gradual decrease in cell viability (Figure 3A). We also used a mitochondrial membrane potential kit (JC-1) to detect changes in mitochondrial membrane potential in cardiac myocytes. The results showed that as HPM increased, Q3/Q2 gradually increased and mitochondrial membrane potential gradually decreased (Figure 3C). Next, the expression of PTGS2 in cardiomyocytes after irradiation with different HPM was measured by western blot and PCR (Figures 3D,F).

### 3.6. Gradual increase in cardiomyocyte ferroptosis with increasing HPM dose

we used flow cytometry to detect intracellular reactive oxygen levels. We found that intracellular ROS gradually increased with increasing HPM (Figure 3B). Using western blot to detect the expression levels of GPX4, HMOX1, ACSL4, SLC7A11, the important proteins in the ferroptosis pathway in cardiac myocytes (Figure 3E).

### 3.7. GPX4 plays an important role in the ferroptosis in HPM-irradiated cardiomyocytes

We knocked down and overexpressed GPX4 in cardiomyocytes and detected GPX4 expression levels using western blot (Figure 3H). After irradiating the knockdown or overexpressed cardiomyocytes with HPM, we found that ROS increased after irradiation in the siGPX4 cardiomyocytes, while ROS decreased after irradiation in the ovGPX4 cardiomyocytes (Figure 3I). Then, we detected the mitochondrial membrane potential with JC-1 kit, and we found that Q3/Q2 increased and mitochondrial membrane potential decreased after irradiation of cells with siGPX4, while Q3/Q2 decreased and mitochondrial membrane potential increased after irradiation of ovGPX4 (Figure 3J).

### 3.8. Tanshinone IIA and Fer-1 protected mice from HPM irradiation injury, as well as significantly reduced myocardial injury

After the administration of tanshinone IIA or Fer-1, the mice showed improved mental status and a significant increase in food and water intake. The mice had increased body weight, decreased heart weight and lung weight, decreased the ratio of heart weight, lung weight and body weight, and decreased the ratio of heart weight, lung weight and tibia length (Table 2).

**Table 2:** Body weight, heart, lung weight tibia length parameters in HPM and treatment group mice.

	Control (*n* = 6)	HPM (*n* = 6)	HPM + Tanshinone (*n* = 6)	HPM + Ferrostatin-1 (*n* = 6)
Body weight (g)	19.75 ± 0.47	17.78 ± 0.41	18.57 ± 0.31	18.95 ± 0.39
Heart weight (mg)	111.52 ± 1.72	184.78 ± 5.15	139.53 ± 3.99	141.82 ± 2.77
Lung weight (mg)	142.48 ± 3.20	227.85 ± 2.57	178.97 ± 3.29	182.85 ± 4.71
Tibial length (mm)	17.35 ± 0.23	17.37 ± 0.37	17.42 ± 0.44	17.57 ± 0.29
Heart weight/body weight	5.65 ± 0.20	10.39 ± 0.25	7.52 ± 0.33	7.49 ± 0.22
Lung weight/body weight	7.22 ± 0.31	12.82 ± 0.31	9.64 ± 0.10	9.65 ± 0.37
Heart weight/tibial length	6.43 ± 0.09	10.65 ± 0.48	8.02 ± 0.32	8.07 ± 0.14
Lung weight/tibial length	8.21 ± 0.17	13.12 ± 0.18	10.28 ± 0.36	10.41 ± 0.31

**Figure 4:**
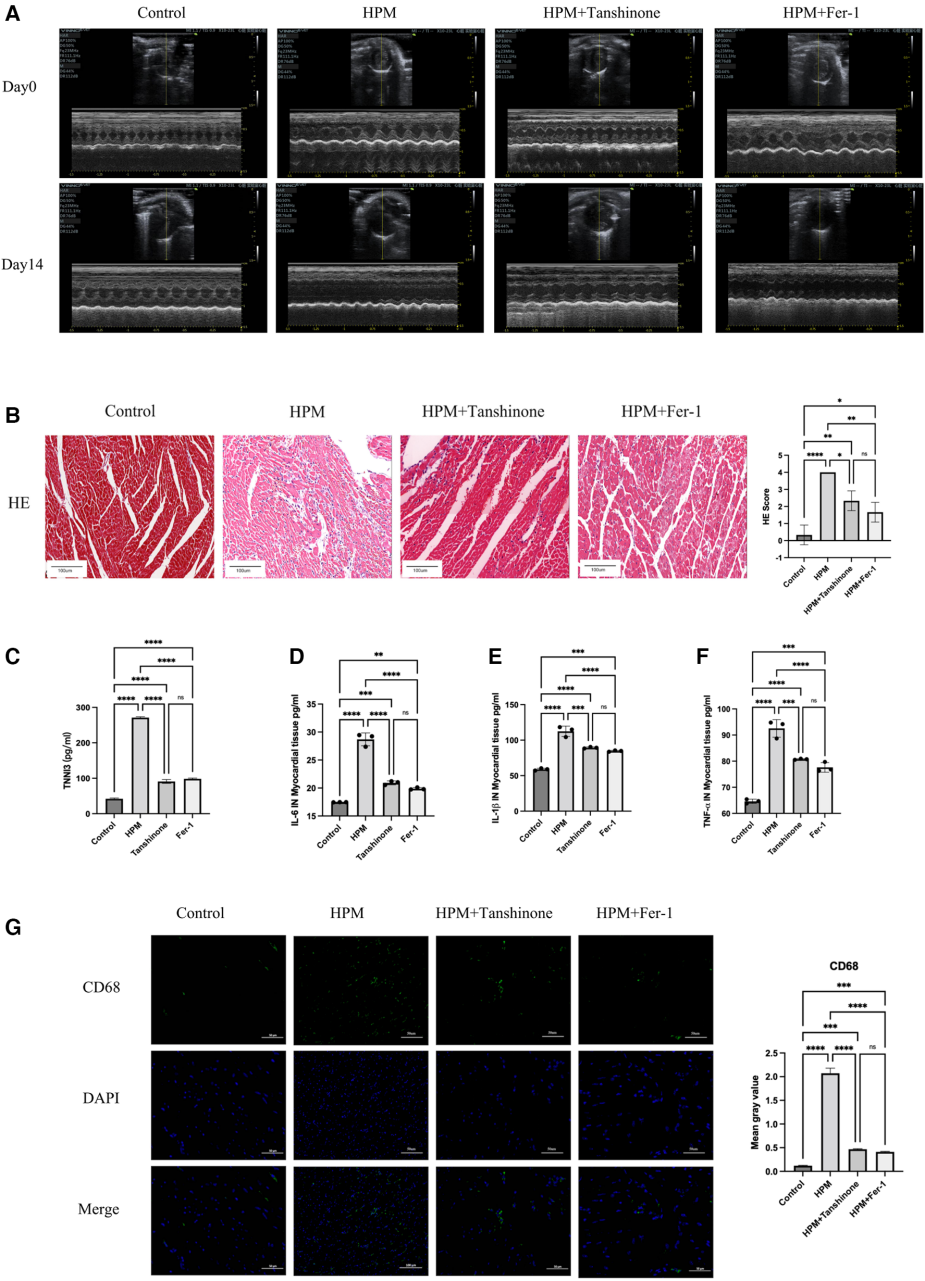
Tanshinone and Fer-1 inhibit inflammatory damage to myocardial tissue. (**A**) Echocardiography inControl, HPM 30 mW/cm^2^, Tanshinone and Fer-1 therapy group mice pre and after HPM. (**B**) HE staining in mouse myocardial tissue. Scale bar: 100 μm. On the right, the number of inflammatory cells per mouse under the microscope is counted. Data presented are mean ± SD of microscopic myocardial injury scores in 3 mice. *, *P* < 0.05; **, *P* < 0.01;****P < 0.0001; ns, not significant, one-way ANOVA, *post hoc* comparisons, Tukey's test. (**C**)TNN3 concentration in mouse serum. (**D–F**) Elisa test the levels of IL-6, IL-1β and TNF-α in myocardial tissue of mice. (**G**) Immunofluorescence technique staining of CD68, DAPI in myocardial section were conducted. Scale bar: 50 μm.

Comparison of pre- and post-HPM echocardiograms from the four groups revealed that the tanshinone and Fer-1 groups compared with the HPM group resulted in increased myocardial contractility, return of EF and FS as well as an increase in LVIDs (Figure 4A and Supplementary Extend Figure S1B).

HE staining shows that the use of the drug resulted in regular myocardial shape and reduced inflammatory cell infiltration in mice (Figure 4B). In addition, HPM-irradiated mice showed reduced myocardial and calprotectin after the administration of tanshinone or Fer-1. And the difference between two drugs was not significant (Figure 4C).

### 3.9. Tanshinone IIA and Fer-1 reduce myocardial inflammation after HPM irradiation in mice

We used Elisa kits for IL-6, IL-1β and TNF-α to measure myocardial inflammation in the HPM irradiated group and in the mice treated with Tanshinone IIA and Fer-1. We found that both Tanshinone and Fer-1 reduced the level of inflammation in the mice, but the difference between the two was not significant (Figures 4D–F). Immunofluorescence testing of CD45 and CD68 expression levels revealed that CD45 and CD68 expression levels were reduced by drug treatment (Figure 4G and Supplementary Extend Figure S1C).

### 3.10. Tanshinone IIA and Fer-1 attenuate myocardial ferroptosis induced by HPM irradiation in mice

**Figure 5:**
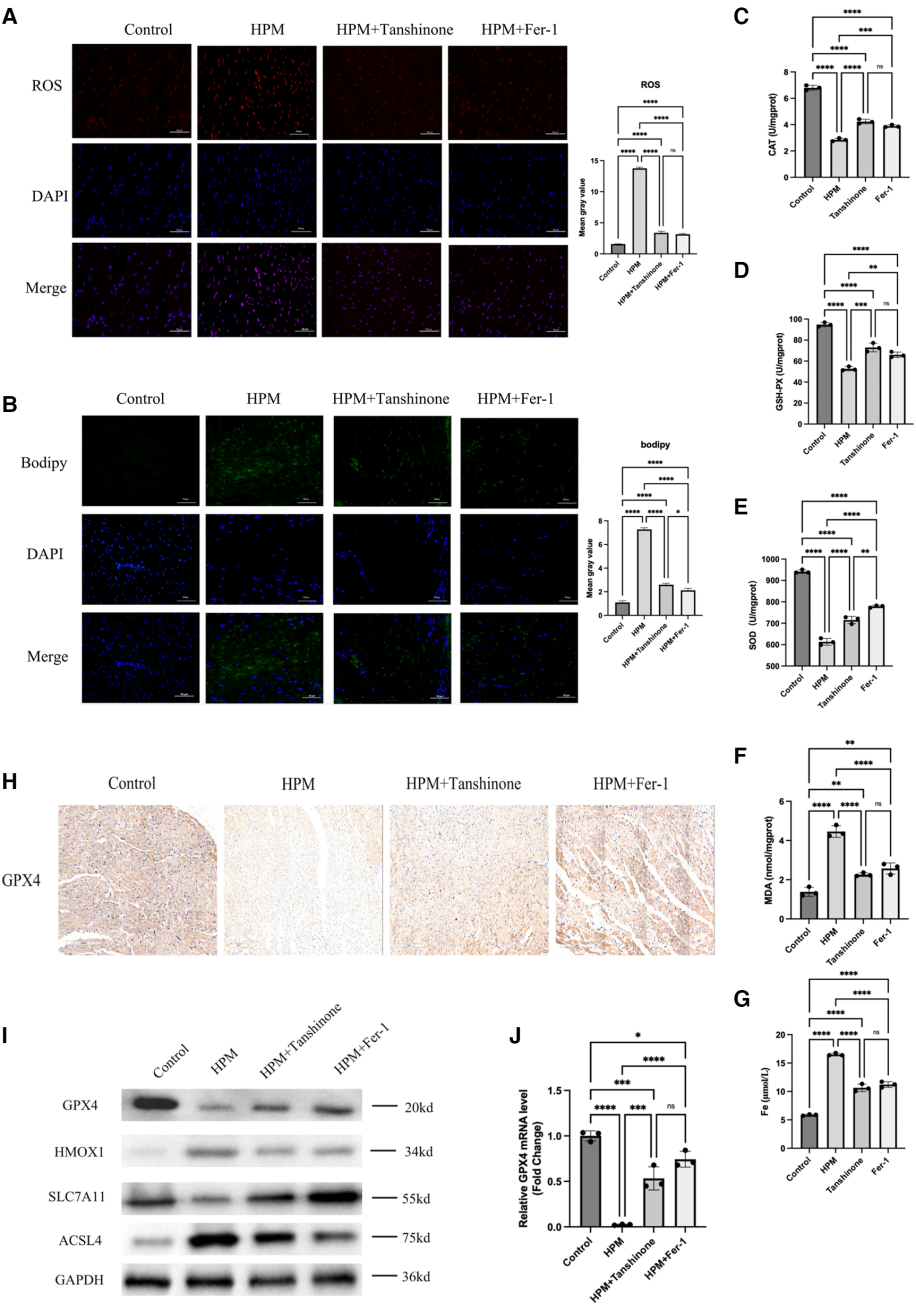
Tanshinone and Fer-1 inhibit ferroptosis in myocardial tissue of mice (*n* = 6). (**A,B**) Immunofluorescence technique staining of ROS, bodipy and DAPI in control, HPM, HPM + Tanshinone and HPM + Fer-1myocardial section were conducted. Scale bar: 50 μm. (**C–F**) Elisa kit detect MAD, CAT, SOD and GSH of myocardial redox status in control and treatment group mice. (**G**) Serum Fe concentration in mice. ****, *P* < 0.0001;***P < 0.001;**, *P* < 0.01; ns, not significantone-way ANOVA, *post hoc* comparisons, Tukey's test. (**I**) Protein levels of GPX4, HMOX1, ACSL4, SLC7A11 from the myocardial tissue in HPM-mice and treatment mice were analyzed with Western blot. (**J**) qRT-PCR analysis of GPX4 mRNA level was done in the myocardial tissue. **, P < 0.01, one-way ANOVA.

Using Elisa kits for MAD, CAT, SOD and GSH to measure the levels of these redox indicators, we found that the use of tanshinone and Fer-1 resulted in an increase in the activity of the three redox enzymes as well as a significant decrease in the oxidation product MDA (Figures 5C–F).

In addition, immunofluorescence assays for ROS, Bodipy staining revealed that Tanshinone and Fer-1 resulted in a reduction in both ROS levels and Bodipy (Figures 5A,B). And we used immunohistochemistry to detect the expression levels of GPX4 and found that the drug could increase the expression levels of GPX4 (Figure 5H). In addition, we examined the expression levels of GPX4, HMOX1, ACSL4 and SLC7A11, important proteins in the ferroptosis pathway, using western blot and found that the drugs increased the expression levels of GPX4 and SLC7A11 as well as decreased the expression of HMOX1 and ACSL4 (Figure 5I).

### 3.11. Tanshinone, Fer-1 reduced HPM irradiation-induced ferroptosis in cardiomyocytes

**Figure 6:**
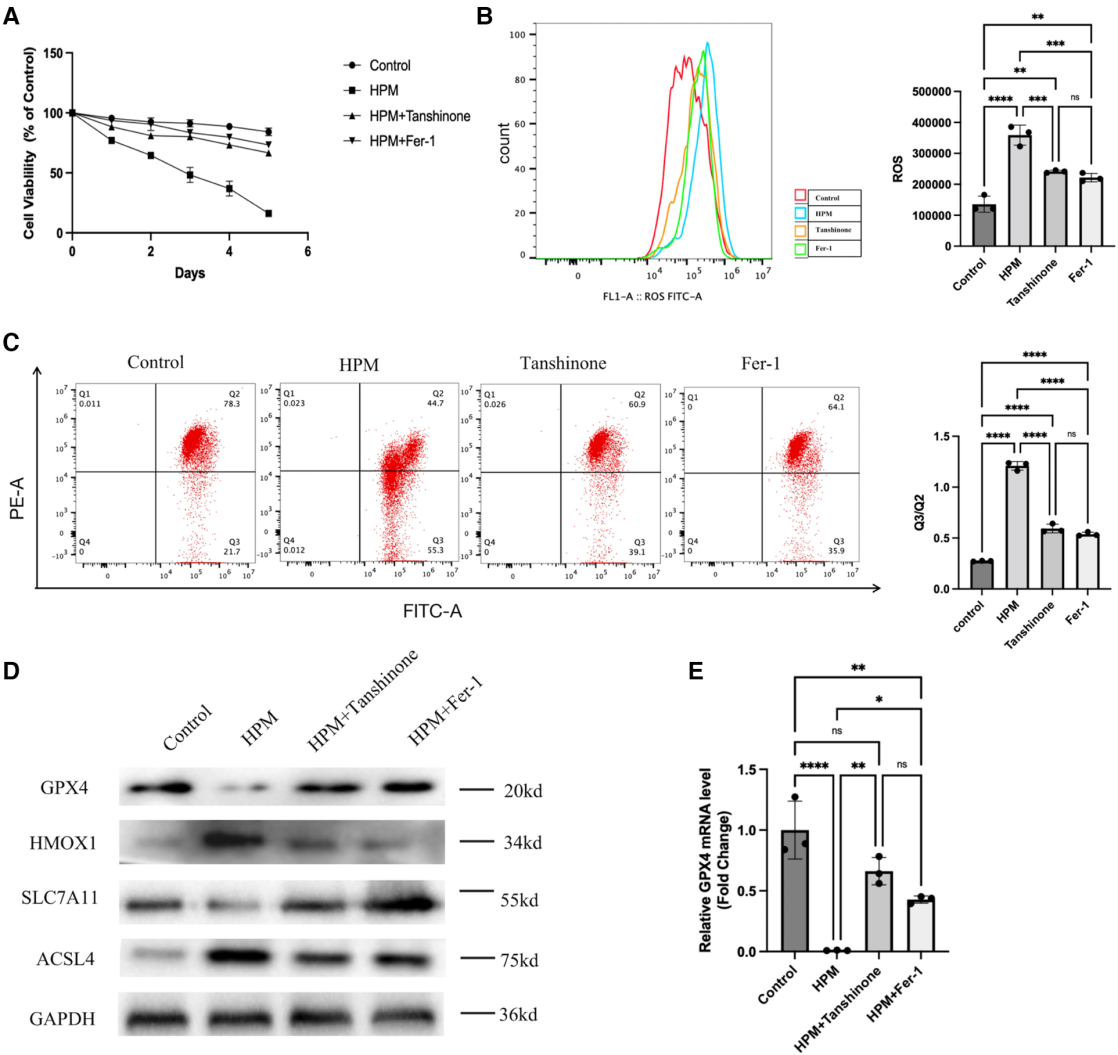
Tanshinone and Fer-1 inhibit ferroptosis in cardiomyocytes. (**A**) Cardiomyocytes cells were treated with Tanshione or Fer-1, and the cell viability was measured by MTS assay from 0 to 5 day after HPM. (**B**) ROS assay was performed in the cardiomyocytes treated with Tanshione or Fer-1. Representative flow cytometry dot plots (left) for cardiomyocytes cells and quantitative analysis (right). Data represent mean ± SD. ns, not significant; **, P < 0.01,***, P < 0.001,****, P < 0.0001, one-way ANOVA, *post hoc* comparisons, Tukey's test. (**C**) JC-1 Mitochondrial Membrane Potential Assay Kit performed in the cardiomyocytes. d)Protein levels of GPX4, HMOX1, ACSL4, SLC7A11 from the cardiomyocytes were analyzed with Western blot. (**E**) qRT-PCR analysis of GPX4 mRNA level was done in the cardiomyocytes. **, P < 0.01, one-way ANOVA.

Cell viability assays using MTS revealed that tanshinone and Fer-1, resulted in a decrease in cardiomyocyte viability (Figure 6A). Flow cytometry assay of ROS levels revealed that the drug significantly reduced the increase in ROS in cardiomyocytes induced by irradiation (Figure 6B). In addition, mitochondrial membrane potential was increased compared to the irradiated group alone (Figure 6C). Using western blot to detect the expression levels of GPX4, HMOX1, ACSL4, and SLC7A11, it was found that the drug reduced the expression levels of iron death-inducing proteins and increased the expression levels of iron death-preventing-related proteins in cardiomyocytes (Figure 6D). And PCR results showed that drug treatment could increase the mRNA levels of GPX4 (Figure 6E).

## 4. Discussion

Previous studies have demonstrated that the heart is one of the target organs for damage caused by microwave radiation (including s- and x-band), causing structural and functional abnormalities (28). However, the mechanisms responsible for myocardial damage have rarely been reported. Herein, we systematically investigate the role of ferroptosis, a newly identified form of iron-dependent regulated cell death, in cardiac injury induced by s-band microwave radiation at different average high powers.

It is well known that the effects of electromagnetic radiation on living organisms are researched in two different mechanisms: the bio-thermal effect and the non-thermal effect. The non-thermal effect focuses on the damage caused by electromagnetic waves in the body when the amount of heat generated is low, the energy is not high and the increase in body temperature is not significant. We found that when the anal temperature of mice was measured immediately after irradiation with different irradiation powers, the increase in temperature did not exceed 0.5°C, which is a non-thermal effect. Therefore, our study provides insight into the non-thermal effects of HPM on myocardial injury.

The major findings from the present research revealed that ferroptosis of cardiomyocytes was activated in mice with microwave irradiation in the S-band and as the irradiation power increases, the degree of ferroptosis in the cardiomyocytes as well as the damage to myocardial tissue and cardiac function, gradually increases. On the contrary, Tanshinone IIA with a low dose at 50nM and ferroptosis inhibitor Ferrostatin-1 improved cardiac function, alleviated inflammation, restored enzymatic antioxidant content, reduced lipid peroxidation products, decreased iron loading, repressed the expression of PTFS2 and ACSL4, and increased the expression of GPX4 and SLC7A11. Furthermore, when GPX4 was knocked down or overexpressed in microwave-irradiated primary cardiomyocytes, we found that cells of viability, cellular mitochondrial membranes potential and cellular production of ROS were different and that the expression of associated ferroptosis proteins were altered quite differently.

Ferroptosis is a result of oxidative phospholipid damage, and a nonapoptotic iron-dependent death pattern. Cells in high polyunsaturated lipid status and increased ROS resulting from increased intracellular iron compliance are susceptible to ferroptosis (29, 30). Lately, new evidence has emerged pointing to an essential function for ferroptosis in the development of cardiomyopathy (31). Ferrrotposis-like cell death occurred in H9C2 myocytes induced by erastin or isoprenaline (ISO) and in a model of heart failure(HF) caused by aortic constriction (32). Ferroptosis is also associated with diabetic myocardial ischemia/reperfusion injury by modulating endoplasmic reticulum stress (33). Furthermore, Fang et al. showed that ferroptosis was involved in DOX and ischemia reperfusion (I/R)-induced cardiac injury (19). Pharmacological inhibition of ferroptosis provides significant protection against the development of cardiomyopathy. In the present research, we found that cardiac as well as myocardial progenitor cells undergo ferroptosis induced by microwave irradiation and that the degree of ferroptosis was positively correlated with the irradiation power. Pharmacological inhibition of ferroptosis significantly alleviated cardiac injury and inflammation, and enhanced cellular viability.

Initially, the advent of mobile telephones, radar, satellites, wireless networks, microwave ovens and GPS devices facilitated our lives, but electromagnetic waves are a double-edged sword and we have to think about the negative effects they have on our organism (34). Cardiac enzymes are specific enzymes which are found which include AST, CK-MB LDH, cTNT. When myocardial cells are injured, cardiac enzymes may be raised for a period of time and may be combined with echocardiographic assessment or other relevant tests to identify the degree and type of damage (35, 36). This research uses serum biochemical analysis and cardiac ultrasound to detect changes in cardiac function in mice after microwave irradiation. The results showed that with the gradual intensification of irradiation power, the mouse heart gradually showed damage and increased AST, LDH, CK-MB and cTNT activities. The above results may be related to the damage of cardiomyocyte membrane structure and alteration of permeability, leading to the entry of cardiac enzymes into the blood. Cardiac injury activates innate immunity and triggers an inflammatory response (37). Macrophages may not be evident in a healthy heart, but the number of macrophages is greatly augmented after myocardial injury (38). We measured IL-6, IL-1β, TNF-α by Elisa, RT-PCR, HE staining and CD45, CD68 by immunofluorescence both of which are leukocytes and macrophages labels respectively. Our results are consistent with previous results in septic cardiomyopathy studies and the inflammatory response due to injury is most severe when the irradiation power is highest (39). Iron overload leads to an overproduction of ROS, which contributes to excessive oxidative stress and lipid peroxidation, due to insufficient antioxidant pathways (40). The main antioxidants in the body include GSH-PX, CAT, SOD, etc. Moreover, MDA is the main aldehyde product of lipid peroxidation (40, 41). In our investigation, we found that with increasing irradiation doses, myocardial tissue iron loading was elevated, while elevated MDA was detected, and associated antioxidants were reduced. Subsequently, we used C11-BODIPY fluorescent staining, a marker of ferroptosis, to monitor the extent of lipid peroxidation (42), thus further validating the presence of iron load-dependent lipid peroxidation, i.e., ferroptosis, during irradiation. Mitochondrial dysfunction and metabolic alterations induced by intra- and extracellular signals determine the destiny of the cell. We further examined Ros by immunofluorescence, flow cytometry and found that the higher the irradiation power the higher the Ros.

Mitochondria play a core role in material, energy and iron metabolism and are the major cell organelles in iron utilization, catabolic and anabolic pathways (43). We used the JC-1, flow cytometry method to detect mitochondrial membrane potential (44), and further reflect the functional state of mitochondria. Similarly, mitochondria do become dysfunctional in response to microwave irradiation. Glutataione(GSH) is widely found in animals and plants and is an antioxidant with the ability to clear free radicals and protect many thiols in proteins and enzymes (45). Cause of ferroptosis include reduced expression of GSH (46). One study found that the GSH content in 661W cells was reduced after microwave radiation (47). GSH acts as a cofactor for GSH peroxidase 4(GPX4) in lipid metabolism. Suppression of GPX4 induces the accumulation of lipid peroxides and ferroptosis (48). Therefore, we suggest that there may be ferroptosis in cardiac with microwave irradiation.

Prostaglandin endoperoxide synthase 2 (PTGS2), also known as cyclooxygenase-2 (cox-2), is a well-established marker of ferroptosis and its increased expression levels have also been observed in the hearts of sepsis mice (49, 50, 51). Acyl-CoA synthetase long chain family member 4 (ACSL4) is critical for apoptosis in iron chain cancer cells. The expression of ACSL4 was significantly decreased in ferroptosis-resistant cells (52). Thus, ACSL4 is involved in the catalysis of polyunsaturated fat oxidation, i.e., in ferroptosis. GPX4 downregulation is often used as a marker for ferroptosis (53–56). GPX4 can capture phospholipid peroxides thereby preventing lipid peroxidation as a protective measure against ferroptosis (57). SLC7A11 is a cystine/glutamate antiporter, specifically expressed on the cell membrane and responsible for maintaining redox homeostasis through the import of cystine (55). In 2012, pharmacological blockade of SLC7A11-mediated cystine uptake by compounds such as erastin was found to induce a new form of cell death called ferroptoasis (58). We tested iron death-related proteins, including ptgs2, GPX4, SLC7A11, ACSL4, *in vivo* and *in vitro* and found that they were expressed differently at different microwave irradiation powers. One study found recently that ferristatin-1 (Fer-1) is a potent and specific inhibitor of ferroptosis (59). In our research, cardiac function, inflammation, and ROS (including ferroptosis-related proteins) were found to be improved by giving Fer-1. These results provided a strong evidence of ferroptosis to participate in cardiac injury in microwave irradiation mice.

GPX4 is the first ferroptosis inhibitor to be discovered and plays a key role in ferroptosis (60, 61). Damage to GPX4 can lead to ferroptosis and overexpression of GPX4 prevents cellular damage due to oxidation (62–64). GPX4 overexpression in mitochondria can inhibit mitochondria-dependent apoptosis as well as oxidative stress in mitochondria (65, 66). To the best of our knowledge, there are no studies related to GPX4 in microwave irradiation myocardial injury. We performed GPX4 knockdown and overexpression in irradiated cardiomyocytes *in vitro* and found that cell viability and mitochondrial membrane stability were optimal when GPX4 was overexpressed. However, when GPX4 was knocked down, ROS production was significantly increased, while cell viability was significantly decreased. GPX4 is also a critical core player in myocardial injury induced by microwave irradiation.

A number of studies have identified natural compounds that can be used to alleviate regulatory ferroptosis and thereby improve pathological conditions (22, 24, 32, 67). TSA is widely used in the treatment of cardiovascular disease because of its ability to regulate intracellular redox functions to protect cells from damage (26). TSA also could act as an anti-atherosclerotic agent by reducing the inflammation associated with cholesterol accumulation (26, 68). TSA can significantly improve endothelial cell function by inhibiting ROS-induced oxidative stress, reducing apoptosis and alleviating hypoxic injury (53, 54, 56, 69). Based on these, researchers have found that TSA can mitigate ferroptosis to protect the corresponding target organs (25, 70). In this research, we further evaluated the effect of TSA on microwave irradiation-induced myocardial injury and ferroptosis to provide a basis for next steps in treatment and prevention.

We could significantly decrease the secretion of proinflammatory cytokines IL-1β, IL-6, and TNF-α (mainly secreted by M1 macrophages)after microwave irradiation using low concentrations of TSA *in vivo* and *in vitro* experiments, respectively. This situation is consistent with following FER-1 inhibition (71). This result may be mainly due to the fact that tanshinone IIA and Fer-1 can reduce the proportion of M1 macrophages to alleviate the inflammatory response after early application of irradiation-induced myocardial injury. In addition, TSA treatment significantly inhibited cell death, improved cardiac function, decreased ROS, MDA production, increased SOD, CAT, and GSH-PX levels, and promoted GPX4, SLC7A11 expression after induction of specific ferroptosis degradation after exposure to irradiation, and decreased the expression of induced ferroptosis associated proteins PTGS2 and ACSL4 in our study. These results suggest that ferroptosis occurs in the myocardium under high power microwave irradiation in the S-band, in which GPX4 remains a key molecule. TSA as well as Fer-1 may play a cardioprotective role by early inhibition of inflammation, reduction of iron loading and reduction of lipid peroxidation.

## 5. Conclusions

In summary, the S-band microwave radiation was followed by abnormal cardiac function, structural damage to the myocardium and reduced viability of cardiomyocytes, suggesting the successful establishment of an S-band microwave radiation heart injury model. Moreover, with the power intensity of microwave irradiation, myocardial tissue as well as cardiomyocytes showed a gradual increase in inflammation, a continuous deterioration in cardiac function. In addition, with increasing irradiation dose, the activity of three redox enzymes: CAT, SOD and GSH-PX decreased in myocardial tissue, while MDA, a product of redox reactions, gradually increased. The level of ROS in myocardial tissue and myocytes increased, Bodipy, which responds to the level of lipid peroxidation, increased, and the mitochondrial membrane potential in myocardial cells gradually decreased. This suggests that the level of ferroptosis, in myocardial tissue and cardiomyocytes gradually increased with increasing HPM. Our study found that the above injury could be alleviated using Fer-1, demonstrating for the first time the presence of ferroptosis in this model injury. Meanwhile, GPX4 continued to play a key central role. The occurrence of ferroptosis in cardiac myocytes can be influenced by altering the expression of GPX4 in cardiac myocytes. The use of low concentrations of TSA inhibited ferroptosis, alleviated myocardial as well as cardiomyocyte functional viability recovery and maintained a positive redox state. This study provides new directions for exploring the mechanisms of cardiac injury after microwave radiation, new applications for the pharmacological mechanisms of TSA, and potential new strategies for the study of injury prevention treatment methods. This research is only a preliminary investigation into the mechanisms of myocardial injury from microwave irradiation and the related injuries treated with Fer-1 and TSA. More in-depth mechanistic studies need to be done by more academic colleagues.

## Data Availability

The raw data supporting the conclusions of this article will be made available by the authors, without undue reservation.
